# Explicating Exact versus Conceptual Replication

**DOI:** 10.1007/s10670-021-00464-z

**Published:** 2021-09-29

**Authors:** Robert Hudson

**Affiliations:** Department of Philosophy, University of Saskatchewan, 9 Campus Drive, Saskatoon, SK S7N 5A5 Canada

## Abstract

What does it mean to replicate an experiment? A distinction is often drawn between ‘exact’ (or ‘direct’) and ‘conceptual’ replication. However, in recent work, Uljana Feest argues that the notion of replication in itself, whether exact or conceptual, is flawed due to the problem of systematic error, and Edouard Machery argues that, although the notion of replication is not flawed, we should nevertheless dispense with the distinction between exact and conceptual replication. My plan in this paper is to defend the value of replication, along with the distinction between exact and conceptual replication, from the critiques of Feest and Machery. To that end, I provide an explication of conceptual replication, and distinguish it from what I call ‘experimental’ replication. On the basis, then, of a tripartite distinction between exact, experimental and conceptual replication, I argue in response to Feest that replication is still informative despite the prospect of systematic error. I also rebut Machery’s claim that conceptual replication is fundamentally confused and wrongly conflates replication and extension, and in turn raise some objections to his own Resampling Account of replication.

## Introduction

It has recently become a pressing concern that many verified scientific, experimental results fail to replicate. But what does it mean to replicate an experiment? A distinction is often drawn between ‘exact’ (or ‘direct’) and ‘conceptual’ replication. On that basis, it is suggested by some methodologists that scientists should strive for exact replications (for example, Cesario, 2014; Hüffmeier et al., 2016; Pashler & Harris, 2012; Simons, 2014), whereas others vouch for the priority of conceptual replications (Crandall & Sherman, 2016; Lynch et al., 2015; Stroebe & Strack, 2014). But Feest (2019) has recently argued that the notion of replication itself, whether exact or conceptual, is flawed (Feest, 2019), whereas Machery (2020) has argued that, although the notion of replication is not flawed, we should dispense nevertheless with the distinction between exact and conceptual replication.

In this paper I plan to defend the value of replication, along with the distinction between exact and conceptual replication, from the critiques of Feest and Machery. Feest argues that the problem of systematic error shows that neither exact nor conceptual replication have much use in scientific, experimental investigation. In response to Feest, I argue that the problem of systematic error is not so dire as to undermine the value of replication, and that, in fact, replication is fundamental to scientific inquiry, even in cases where one is investigating the occurrence of systematic error. For his part, Machery claims that conceptual replication is fundamentally confused—for example, it conflates the notions of a replication and an extension—and argues that the only meaning of a replication left to be considered is captured by his ‘Resampling Account’. In response to Machery, I provide an improved analysis of conceptual replication that distinguishes it from what I call ‘experimental’ replication, where both these forms of replication are themselves distinct from exact replication. On the basis of this tripartite distinction, I then explain how Machery’s objections to conceptual replication can be disputed, while his own Resampling Account is vulnerable to conflating, once again, replication and extension. Overall, then, the value of replication is reaffirmed, contra the view of Feest, and the distinction between exact and conceptual replication is reaffirmed as well, contra the view of Machery.

## Exact versus Conceptual Replication

A scientist runs an experiment and generates a result R. Was R just a fluke or was it an inevitable result of experimental design? To determine this, the scientist runs the experiment again, keeping everything the same as far as possible. Loosely speaking, this is what is called an ‘exact replication’.

It is often complained that an exact replication is impossible since there will inevitably be at least some subtle changes, at least in a temporal sense, from the initial running of the experiment (see Lynch et al., 2015, 333; Stroebe & Strack, 2014, 67). Machery has an effective response to this problem (2020, 549), suggesting that any judgment about the sameness or difference of a sequence of events, such as an experiment, is relative to some criterion that specifies when a sequence is the same as or different from another sequence. Thus, in attempting to perform an exact replication of an experiment, one need not ensure that the subsequent experiment is the same as the original one in all respects—only those respects relevant to the suggested criterion matter. So, for example, an experimenter may judge that a subsequent run of experiment at 3:00 pm is an exact replication of an original experiment run at 2:00 pm since she adopts a criterion of sameness in which time of day is viewed as irrelevant. It is also often complained that, even if one can perform exact replications, such replications are ultimately uninformative. For even if we grant that a certain result inevitably comes about with every exact replication of experiment, all we’ve shown is that this result is produced in this unique situation, without any implication that the result is preserved under alternate scenarios (on this objection, see Lynch et al., 2015, 335; Crandall & Sherman, 2016, 95). In this sort of circumstance it is nevertheless recognized, even by those who reject the value of exact replications generally speaking, that such replications have significant value. For example, Stroebe and Strack note the importance of exact replication in cases where “the scientist... wants to establish the efficiency of a specific treatment or intervention”, as opposed to an inquiry in “basic research where empirical outcomes are meaningful only with respect to the theory being tested” (2014, 60). Similarly, Crandall and Sherman comment that “[t]here are many cases in which careful attention to exact replication is essential,... especially [where this] might lead to policy recommendations” (2016, 97; see also Schmidt, 2009, 97, about “unconventional claims”, and Hüffmeier et al., 2016, 83, concerning “truly new findings”). Thus, Pashler and Harris note that the discovery of cold fusion by Pons and Fleischmann in the 1990s prompted a spate of exact replication attempts (2012, 534), notwithstanding apparent misgivings about the presumed impossibility of ever truly performing an exact replication. It is also suggested by Cesario (2014) that exact replications have a value both in determining the size of a observed effect, as well as providing an indicator of whether an effect is actually the product of a type one error (41). Moreover, Cesario suggests that if an experimental process, after being changed and not exactly replicated, fails to detect an originally discovered effect, one has the option of identifying and attributing this failure to a ‘moderating factor’. He thus requires that exact replications of an experimental process be performed in the same research lab to minimize this possibility (2014, 44). Other methodologists, by comparison, are less strict on this point. Simons (2014) responds directly to Cesario by requiring replications to at least be performed in different labs, thus ensuring that a generated effect is not due to some idiosyncratic feature of the original laboratory experiment, one unrelated to the reality of the effect (77). In this respect, Hüffmeier et al. (2016) distinguish between ‘exact’ and ‘close’ replications, the latter being a case where the same experimental process is performed, to whatever degree possible, by independent researchers (84). But again the point is made by Hüffmeier et al. that close replications, so defined, are needed to address the issues of determining effect sizes and recognizing type one errors.

So from the above we can conclude that exact replications are, after all, performable and can be informative. However they have a more serious problem, often noted: it is possible for an experimental process to generate an effect, and to do so with exact replications, but for this effect to be nevertheless illusory. Crandall and Sherman cite the case of research on cognitive dissonance found in Brehm (1956) which isolated a statistical effect “directly replicated many times in different labs, across different decades, and using different kinds of subjects” (95), but which was, it turns out, misleading due to a flaw in experimental design, a flaw recurring in every direct replication. Feest makes a similar point, arguing that direct replication is susceptible to the problem of systematic error “since there is always a possibility of overlooked confounding variables” (2019, 902). A common answer to this problem is to recommend a different sort of replication, one that varies the circumstances of the experimental process in targeted ways so as to reveal the presence of such variables. The name often given to this sort of replication is ‘conceptual’ replication. As Lynch et al. describe this form of replication (which they also call ‘robustness’), one replicates the “independent and dependent variables [utilized in an experiment] with operationalizations that vary in multiple ways from the original [experimental process]” (2015, 335). Here they cite Cook and Campbell (1979) who call this strategy “deliberate sampling for heterogeneity” (76). Similarly, conceptual replication for Crandall and Sherman occurs when predicted experimental effects continue to hold “across a range of operationalizations of independent and dependent variables” (2016, 95). One finds analogous understandings of conceptual replication in Schmidt (2009, 93), Stroebe and Strack (2014, 62–63), Lynch et al., (2015, 335), Feest (2019, 900), and elsewhere. The idea, then, is that with exact or close replication one seeks to determine whether the same effect occurs when an experiment is repeated given a particular operationalization of a set of dependent and independent variables, whereas with conceptual replication one alters the operationalization of the dependent and independent variables and then determines, on that basis, whether the same effect comes about.

From here, one can perform a conceptual replication in two ways. On the first approach, one retains the variables under consideration and formulates different operationalizations for them. This is seemingly what is being suggested above with Crandall and Sherman (2016), Lynch et al. (2015), and the others. Alternatively, one can perform a conceptual replication by formulating entirely different kinds of variables and then operationalizing accordingly. Note that in this latter sort of situation the variables will, in all likelihood, lead to different operationalizations (in the unlikely situation where these different variables are represented by the same operationalization, the operationalizations would nevertheless mean something very different). To provide an illustration of the first kind of conceptual replication, Stroebe and Strack consider an experimental test of a thesis in the social psychology of persuasion, that “the impact of the quality of the arguments contained in a communication is greater the more thoughtfully and deeply the communication is processed by a recipient” (2014, 63). Here, Stroebe and Strack note, the dependent variable in this thesis, the degree to which someone processes a communication “thoughtfully and deeply”, can be operationalized in terms of “distraction” or “personal relevance” or “expectation to have to discuss the communication at a future meeting” or “the need for closure”. As such, different experiments can be performed that are conceptual replications of one another utilizing these different operationalizations (see 63 for citations). With this kind of conceptual replication, one simply empirically represents the same dependent variable by means of different operationalizations. To give an example of the second kind of conceptual replication, consider a case taken from research on behavioral priming. Cesario (2104) discusses an experiment from Bargh et al. (1996) seeking to establish whether individuals, when subliminally primed with pictures of Black males as opposed to White males, exhibit “more aggressive responses to provocation” (42). A conceptual replication of this experiment, according to Bargh (2012), studies the effects of priming subjects with words indicative of elderly stereotypes on the tendency of these subjects to subsequently walk more slowly (see Cesario, 2014, 45). Here one is dealing with different kinds of variables altogether: being primed with pictures of Black males is completely unrelated to words indicative of elderly stereotypes, just as exhibiting aggressive behavior is unrelated to walking slowly. It is in this respect that the two experiments involve different operationalizations: they are not different operationalizations of the same variables but rather different operationalizations resulting from the use of different variables altogether.

Thus, whereas with the first type of conceptual replication we keep the variables the same and just try out different operationalizations, with the second type we alter the variables themselves. As such, in the second case, one might resist calling the new experiment a replication of the first. However, such experiments can be considered replications of one another if they are designed to test the same theoretical construct, which is what Bargh is suggesting as regards the theoretical construct ‘priming’. Definitions of conceptual replication suggested by methodologists often make note of this further point, going beyond the initial innovation of using alternate operationalizations. For example, Schmidt (2009) comments:

conceptual replication reaches further than a direct one. The successful replication of a hypothesis validates this hypothesis but it also corroborates the theory behind it. In the end, this hypothesis has been tested by two different experimental ideas. Both ideas are derived from the same underlying theory and thus this theory is also confirmed. (95)

Similarly, according to Hüffmeier et al., with conceptual replication,

comparability to the original study is aspired to only in the aspects that are deemed theoretically relevant ... Most if not all aspects [of an original study] may differ [with a conceptually replicated study] as long as the theoretical processes that have been studied or at least invoked in the original study are also covered in a conceptual replication study (2016, 87).

As a result, in the spirit of terminological regimentation, I propose to borrow the definition of ‘conceptual replication’ formulated by Henry Roediger, one that makes the ‘theory’ focus of such replication more explicit:

a conceptual replication tries to replicate the existence of a concept... by using a different paradigm (Roediger, 2012).

This is, in one sense, a very natural definition of conceptual replication: with conceptual replication one is replicating *concepts*. By comparison, one is replicating *experiments* when one simply changes how one operationalizes variables in an experimental design, where the variables are themselves unchanged. As such, I propose to call the latter ‘experimental’ replication. Exact or direct replication is thus a special case of experimental replication in which all aspects of experiment, including the operationalizations, remain the same.

It is important to be clear, though, that with experimental replications where the variables are operationalized differently there is still a sense in which we are dealing with replicated concepts. So, for example, when we differently operationalize the dependent variable that indicates the degree to which someone processes a communication “thoughtfully and deeply” as either “distraction” or “personal relevance” or “expectation to have to discuss the communication at a future meeting”, these operationalizations can be said to fall under the concept of processing a communication “thoughtfully and deeply”. With experimental replications, it is expected that these alternate operationalizations will be easy to specify and largely uncontroversial. One wants to determine whether a phenomenon continues to appear despite changes in how it is revealed by means of an operationalization, without there being much debate over whether or not we truly have an alternate operationalization. By comparison, with a conceptual replication as I am defining it, one is going up a level and altering the variables themselves, not just modifying operationalizations. It is thus a more speculative move, one that requires a theoretical motivation.

This is what we mean in saying, following Schmidt (2009), that a successful conceptual replication of a hypothesis “validates this hypothesis but it also corroborates the theory behind it”, or following Hüffmeier et al. (2016), that “the theoretical processes...invoked in the original study are also covered in a conceptual replication”, or with Roediger (2012) that conceptual replication deals with a “different paradigm”. In effect, a conceptual replication makes a speculative leap that connects two or more distinct variables under the auspices of a theoretical idea. On this basis, we can say why conceptual replications are important for scientific investigation. One of the goals of scientific theorizing is to locate theoretical linkages between seemingly disparate empirical phenomena. Scientists seek fundamental, lawful processes connecting such phenomena in order to provide them with a unified understanding. But in order to find such laws one needs the ability to recognize the presence of the relevant theoretical ideas in differing phenomena. For example, one could not achieve adequate theoretical, scientific understanding of priming if empirical data concerning priming only occurred with pictures of Black males, and we couldn't make the connection to occurrences of priming in unrelated contexts, such as when dealing with words indicative of elderly stereotypes. It is conceptual replication that provides the basis for a unified theoretical understanding of these phenomena by revealing the presence of a theoretical construct in different sorts of experimental scenarios. Such replication is, therefore, fundamental to scientific, experimental investigation.

To further clarify the distinction between conceptual and experimental (including exact) replication, consider an experiment recounted in Schmidt (2017, 237; see also Schmidt, 2009, 94) illustrating and testing the concept of an ‘expectation effect’. In the experiment, research subjects investigating the rate at which rats can navigate a maze are told in advance that some rats are ‘maze bright’ whereas others are ‘maze dull’, indicating their innate respective abilities to complete a maze, even though there are actually no relevant differences between the rats. As it turns out, the subjects recorded observations indicating that so-called maze bright rats completed the maze in shorter times—an ‘expectation effect’. From here one can perform an exact replication, running the same experiment again as closely as possible to its original run, or along the lines of our new terminology, perform an experimental replication by using a different strain of rats or a different kind of maze. Alternatively, Schmidt states, “one [can] also invent another experimental idea to test the general assumption that the [subject’s] expectation had an effect on the results” (2017, 237). Here he describes an experiment investigating the effects of caffeine consumption on the heart rates of human research subjects, where the experimenters are misled into thinking that the research subjects have ingested caffeine when they have not. If we suppose that the experimenters mistakenly observe increases in heart rate, even when caffeine is not ingested, we have a conceptual replication of the original rats running-the-maze experiment. This is because the concept of an ‘expectation effect’ is replicated, though with an entirely different experimental setup involving different operationalizations testing entirely different variables involving humans drinking coffee instead of rats running mazes. Rats running a maze have nothing to do with humans drinking coffee—they are different kinds of physical phenomena involving different kinds of variables. Still, these phenomena can serve to exemplify the same theoretical construct (or concept), what is called an ‘expectation effect’.

Crandall and Sherman capture a similar notion of conceptual replication. As they comment, with conceptual replication,

*ideas* are the unit of analysis. ... The question becomes not whether a specific finding may hold, but whether a theory can be retained in the face of multiple and variable tests of its hypotheses (2016, 95; their italics).

In other words, with conceptual replication one conducts a new experiment that tests the occurrence of a particular theoretical construct or ‘idea’ using different experimental variables. As a further example of this form of replication, Crandall and Sherman describe an experiment examining how the “theoretical notion... [of] political conservatism... comes directly from the architecture of cognition” (2016, 95). Here the concept of “a simple and basic mode of thought” is represented (or conceptually replicated) in four different ways (or by four different variables), of which two ways are by reference to (1) high blood alcohol content and (2) cognitive load through a simultaneous listening task. Either way, one is engaging in a simple and basic mode of thought, even though being drunk is a much different thing, a different variable, than having to think about two things at once. These are different kinds of physical phenomena, and experimenting on them utilizes different operationalizations, even though the overall experimental design is focused on one thing, a simple and basic mode of thought.

Apart from exact, experimental and conceptual replications, there are a variety of other notions of replication one might consider (Schmidt, 2009, 91, has an extensive list). For example, Roediger (2012) describes ‘systematic’ replication as “an attempt to obtain the same finding, but under somewhat different conditions (say, in a memory experiment with a different set of materials or a different type of test)”. An analogous notion Schmidt (2009) calls a ‘follow-up study’ which “combines direct replication with new elements... and has the function of demonstrating that the same results as shown in the original study can be attained with the new setup” (96–97; Schmidt also talks about ‘systematic replications’, a more complicated notion deriving from Hendrick, 1991). One finds a similar notion in Hüffmeier et al., (2016, 86), who call it ‘constructive’ replication and Lynch et al., (2015, 333) with their notion of an ‘extension’. These alternate notions of a replication I would include under the rubric of an ‘experimental’ replication since they all involve alternate operationalizations of the relevant concepts, but do not strive to examine entirely new theoretical constructs (or new concepts, or ideas), as I have defined conceptual replication above.

It is often argued that conceptual (and experimental) replication is more informative than exact replication, for the simple reason that with conceptual (and experimental) replication one is performing different kinds of experiments, with variables operationalized in different ways, and not just repeating the same experiment over and over again, as with exact replication. But whether this is so is largely dependent on the purposes of experimentation. If one wants to be sure about the existence of an effect, that it can be counted on to occur again (leaving aside the more difficult question of whether one’s understanding of the effect is accurate), then exact replication is more informative. Alternatively, if one wants to demonstrate that the same effect comes about with different operationalizations of a certain set of variables and is not simply the result of one particular operationalization, then an experimental replication is more informative. Finally, if one is attempting demonstrate that a theoretical concept applies in different experimental scenarios, then conceptual replication is more informative. These sorts of issues, at any rate, are not what I plan to explore here. Rather, my goal is to defend the epistemic value of replication itself, whether exact or conceptual, from the critiques of Feest (2019), as well as to defend the sustainability of the distinction between exact and conceptual replication from the objections of Machery (2020).

## Feest versus Exact and Conceptual Replication

Feest’s definition of conceptual replication follows closely along the lines of our preliminary definition above. For her, “conceptual replications try to operationalize the same question or concept/effect in a different way” (898), whereas exact, or direct replications duplicate an experiment using the same operationalizations. On her view, there's no question that one is able to perform either direct or conceptual replications. The problem, rather, with both kinds of replication is that they are inevitably afflicted with forms of systematic error, especially in those scientific research areas, such as psychology, that involve a great deal of ‘conceptual openness’ or a ‘high degree of epistemic uncertainty’ (2019, 903–904). Notably, one can successfully directly replicate an effect, but still make a mistake concerning one's interpretation of this effect (for example, in terms of what phenomenon one takes the effect to represent). This is what occurred in the research, mentioned above, performed by Brehm (1956) on cognitive dissonance, which was replicated many times even though “there was an important flaw in the design of the study that compromised... the interpretation of the results” (Crandall & Sherman, 2016, 95). Conceptual replication is often thought to be a strategy one can use to remedy this flaw in direct replication (see Stroebe & Strack, 2014, 62–63; citing Schmidt, 2009, 93; Crandall & Sherman, 2016, 95). But not according to Feest. She says,

if I want to compare the results of two experiments that operationalize the same construct differently, I already have to presuppose that both operationalizations in fact have the same conceptual scope, that is, that they in fact individuate the same effect (2019, 901).

“But”, she continues, “this would be begging the question, since after all—given the epistemic uncertainty and conceptual openness [of a scientific field]—that is precisely what is at issue” (901). Put another way, I can have two different operationalizations of a ‘construct’ (Feest’s term) only if I know what this construct is in the first place, which is what I am trying to discover by performing experiments with these different operationalizations.^1^ More generally, the problem Feest focuses on is that, even with the successful conceptual replication of an effect, in which the same effect comes about despite different operationalizations of the variables, it still might be the case that we are misled in terms of our understanding of this effect, that is, in terms of our understanding of the (psychological) phenomenon that underlies the effect. Agreement on what the effect is, despite differences in how the variables are operationalized, does not guarantee that we have not misconceived the effect. As Feest suggests, to argue robustly that the presence of a “high correlation between the results of two experiments indicates that they operationalize the same concept... is a post hoc judgment and not something that can be taken for granted up front” (901, footnote 5). In other words, a successful conceptual replication could be the result of a systematic error, which undermines the value of such a replication.

How effective is Feest’s critique of conceptual replication? Is it the case that to have a successful, conceptual replication one needs, not only “well-formed concepts... operationalized in different ways”, but a research domain that is already “well understood” (903) and researchers possessing “a relatively good grasp of the relevant concepts” (904)? Feest is correct that performing either exact or conceptual replications that are informative cannot take place in a knowledge vacuum: some understanding of the subject matter at hand must be assumed, and can be expected to inform the strategies experimenters use in attempting to replicate results. In this respect, the difference between exact, experimental and conceptual replications relates to the comparative ambitions of the experimenters. The goal of an exact replication is modest, as one is simply trying to generate the same experimental result given identical experimental conditions, so far as this is possible. Experimental replications, as I have defined this notion, are ambitious in that the goal is to reproduce a result under circumstances in which the dependent and independent variables are operationalized in novel ways. Finally, what I am calling a conceptual replication is substantially more forward-looking. Here a concept is expressed as distinct kinds of variables, which are then operationalized in different ways. With all three kinds of replication, there is a requirement that the investigator has a certain degree of understanding of the phenomenon under investigation. Productive scientific research can ask for no less. Nevertheless, there is no requirement that the investigator have a full understanding of the subject matter. So, for example, in performing an exact replication there is a presumed understanding of the sorts of environmental conditions that are fundamentally irrelevant to the retrieval of a result, and so changing these conditions should permit an exact replication. But the investigator in performing an exact replication in this circumstance does not ‘beg the question’ regarding whether their assumptions concerning these environmental conditions are in fact the case. This is simply because in altering these conditions it may turn out that the attempted replication fails, by means of which the experimenter learns that their previous theoretical assumptions about these conditions was mistaken. Similar comments apply to both experimental and conceptual replication (as I have defined them). Focusing on conceptual replication, experimenters often take a substantive theoretical leap, such as supposing that expectation effects regarding, first, mice running through mazes, and second, humans drinking coffee, are related psychological phenomena. If it turns out that an experiment is successfully conceptually replicated, the relevant theoretical presupposition is confirmed. But the experimenter has not thereby assumed what she’s trying to prove, since the experiment might have turned out otherwise. To appreciate this response, the assertion that one experiment replicates another experiment must occur in advance of the results of the experiments (that is, the suggested replication is ‘pre-registered’). In this way, we avoid making a ‘post hoc’ judgment that ‘begs the question’.

So what are we to make of problem is Feest raising? Her concern seems to be that the effects discovered through conceptual replications are possibly false due to the prospect of the presence of systematic errors, just as exactly (or experimentally) replicated effects are possibly false for the same reason. This is no doubt the case given the fallibility of (especially, for her, psychological) science. Given this fallibility, effects can be found to occur by means of replications, even though these effects are illusory indicating, to this degree, that the effect is not “well-understood” and that one lacks “a relatively good grasp of the relevant concepts”. But the mere fact of this possibility does not undermine those cases of replication where one does have a good understanding of the phenomenon being investigated. Moreover, if Feest’s concern is that we need procedures to investigate the possibility of systematic errors in replication, then attempting replications is a good way to satisfy this need. An experimenter tests the hypothesis that, on their understanding of the concepts involved, an attempted replication will succeed, and if it doesn't succeed in a way that is explicable by means of random error, then they will have discovered a systematic error in their understanding. Our assessment here applies as well to her argument against exact replication. She argues that exact replication, just like conceptual replication, is subject to systematic error. However, that doesn't stop exact replications from occurring (assuming we believe exact replications to be possible, in the first place), nor does it stop exact replications from being informative, for example, if we want confirmation about the occurrence or size of an effect, nor does it stop exact replications from sometimes failing, thus allowing us to diagnose the occurrence of a systematic error.

In general, Feest’s assessment of the value of replication is modest: she wants only to “conclude that replications are less useful and important than is widely assumed” (904; she continues, “at least in the kind of psychological research I have focused on in this article”). But I think we can go further than her and say, not only that conceptual and exact replications are possible (despite their fallibility), but that they are necessary for scientific research. Feest’s view is that, with the demise of both conceptual and exact replication induced by the prospect of systematic error, we need an alternate understanding of experimental methodology. Specifically, since it is systematic error that causes problems with replication, what scientists should do is turn their attention to “exploring, and experimentally testing, hypotheses about possible systematic errors in experiments” (2019, 903). By this means, we can gain a good understanding of the effects we are examining, bringing to light the “unstated auxiliary assumptions pertaining to the conceptual scope of the effect in question” (902) and contributing to our conceptual development “by helping to explore and fine-tune the shape and scope of proposed or existing concepts” (903). Yet Feest’s proposed investigative strategy won't work unless scientists have, at the very least, a set of exact replications they can perennially rely on. Imagine, for example, a case where we are examining the phenomenon of an expectation effect by misleading a researcher into thinking that a research subject has ingested caffeine when, in fact, he has not. Feest is concerned about the prospect of systematic error in such a case, and along these lines I suggest it would be a good recommendation for us to pay close attention to the empirical data available showing that a researcher has, indeed, been misled. Such empirical data might involve the researcher *saying* that the research subject has ingested caffeine, might involve the researcher *writing* this fact down in the notebook, and similar sorts of observable events, where the researcher is mistakenly observing increases in the heart rates of research subjects. Now, in evaluating a running of the experiment, we need to make sure that a researcher did in fact *say* that the research subject ingested caffeine, did in fact *write* this fact down, and so on—and to this end we might check a video recording of the researcher’s utterances or inspect the researcher’s inscriptions in her notebook. But for this experimental ‘quality check’ to be useful, it is necessary that the video is the same each time we check and that each time we inspect the researcher inscriptions, they haven't changed either. This is because if the videos and inscriptions aren't the same, we won’t be able to find correlations between a researcher *saying* something or *writing* something down and further mistakenly observing increases in heart rate. In other words, for Feest’s prospective investigation into the possible systematic errors in an experimental process to get off the ground in the first place, it is necessary that certain exact replications are established, and can be counted on recurring. Analogously, it is not hard to imagine situations where, in order for an inquiry into systematic errors to take place, certain other kinds of replications—close, systematic, constructive, conceptual, and so on—need to hold as well. In all cases of scientific investigation, we need a stable subject matter from which to work, as a reference point from which to consider the effects of suggested environmental or conceptual changes. For this reason, Feest’s argument that we should refocus experimental investigation on the matter of correcting for systematic errors, and concern ourselves less with exact or conceptual replications, fails not just because (1) successful replications do not beg the question concerning whether operationalizations are accurate and, in fact, (2) exact or conceptual replications can be used to diagnose the presence of systematic errors, so long as what constitutes a replication is pre-specified (that is, it is not a ‘post hoc’ judgement), but also because (3) in order to investigate the presence of systematic errors in the first place, one needs to have at one’s disposal exact replications (at the very least) to begin with.

## Machery versus Conceptual Replication

Machery (2020) defends what he calls the Resampling Account of replication according to which “a replication is an experiment that resamples the experimental components of an experiment that are treated as random factors”, where an ‘experimental component’ of an experiment is any “aspect of an experiment that can be independently modified” (557). Here, Machery distinguishes four kinds of experimental components: (1) experimental units, (2) treatments (independent variables), (3) measurements (dependent variables) and (4) settings. Experimental units are the things on which we are experimenting, which in psychological research are often people. And put loosely, what we do in an experiment is treat these units in various ways and measure what we they do, according to a certain experimental regimen.

Put more strictly, an experiment is a sequence of events, where each event involves a subset of the set of experimental units being treated and measured in a particular setting. For definiteness, scientists specify precisely what counts as the set of experimental units from which they are ‘sampling’, that is, what ‘population’ they are sampling from. In this regard, Machery rightfully notes that scientists can be seen as also precisely specifying a set of treatments, a set of measurements and a set of settings, all from which they sample. So, when running an experiment, scientists may not only be sampling from a set of experimental units; they can also sample from the sets of treatments, measurements and settings. What does it mean to sample from, for example, a set of treatments? To illustrate, Machery focuses on an case taken from psycholinguistics, specifically research by Gigerenzer and Hoffrage (1995) exploring the question whether research participants are better able to perform Bayesian inferences dependent on how a probability problem (what Machery calls a ‘vignette’) is formatted. In their experimental work, Gigerenzer and Hoffrage formulate 15 different Bayesian probability problems, each of which can be formatted in four different ways, and what they discover is that the ability of a research participant to solve a Bayesian probability problem depends on how the problem is formatted—the participants are much more successful in solving these problems “in frequency formats” (684). On Machery’s understanding of this experiment, the experimenters sample from the set of experimental units by recruiting 60 participants taken to be representative of this set. They also sample from the set of treatments by exposing the 60 participants to 60 different stimuli (15 probability problems formatted in 4 different ways), where the stimuli are also taken to be representative of the total set of possible treatments (which would include, at least, a vast number of alternate probability problems). Here, Machery acknowledges that the terms of reference for the complete set of treatments is somewhat vague, but asserts that a similar vagueness afflicts the issue of what constitutes a complete set of experimental units (554).

The above example illustrates how an experimenter can sample from a set of treatments, as well as from a set of experimental units. The same possibility exists for measurements and settings. In all these sorts of cases the experimental components are described as ‘random factors’: an experimenter could have drawn a different sample from the total population of experimental units, treatments, and so on. For instance, the 60 participants recruited in Gigerenzer and Hoffrage’s experiment could have been different, within the criteria of whom to include in the total population of experimental units (acknowledged again to be somewhat imprecise), just as the particular set of treatments used in the experiment, drawn from the total set of treatments, could have been different. The goal, then, is to use the retrieved sample to generalize by means of a statistical inference to the entire population of experimental units or treatments, the bulk of which remains unobserved. For Machery, then, an experiment is a replication of another experiment if it samples again, or ‘resamples’, from the original populations corresponding to those experimental components that are random factors. This is Machery’s Resampling Account of replication (557).

By contrast, when experimental components are fixed factors, there is no unobserved population about which one generalizes, given the experimental data. Rather, for fixed factors, one limits oneself to the experimental components as they were observed, and there is no statistical generalization to a larger population. In this sort of case, Machery considers a situation where one is examining the effectiveness of a particular new drug, where one does not aspire to generalize to other sorts of drugs (552). In this respect, there is no possibility of a sampling error, of making a faulty inference to a more general set of components, as there might be with a random factor. So, for example, if in the above experiment from the Gigerenzer and Hoffrage (1995) the experimenter decides to focus on one particular probability problem, formatted in a particular way, to test how well the recruited participants solved this problem, this would be to regard the treatment as a fixed factor, while treating the experimental units as a random factor.

Arguably, designating an experimental component as a ‘fixed’ factor is a misnomer if this means that the component has an observed, singular designation, since there is no implication that the factor is unchangeable. The relevant point is that, if a fixed component is changed, we no longer have a replication of an experiment. Moreover, designating an experimental component as a random factor, as opposed to a fixed factor, is arguably a misnomer as well since, if we change the population of entities associated with a random factor, then once more we no longer have a replication of an experiment. So, perhaps, it might be better to say that if an experimental component is a fixed factor then it is ‘complete’ or ‘completely observed', whereas if a component is a random factor then it is ‘incomplete’ or ‘incompletely observed’. This clarification becomes important in our discussion below of Machery’s distinction between a replication and an extension.

In addition, where we are sampling from a component that is a random factor, what we are doing is introducing alternate operationalizations of an experimental component. For example, in Gigerenzer and Hoffrage’s experiment, each of the 15 Bayesian probability problems they introduce are alternate operationalizations of the treatment of being exposed to a Bayesian probability problem, just as each of the four formatting methods alternately operationalize the treatment of having a certain formatting. Thus, using my terminology, Gigerenzer and Hoffrage provide us with 60 experimental replications of the same experiment.

The replications are not exact replications, given the differences in operationalizations. But nor are they conceptual replications (on my terminology), since they are dealing with the same experimental components: specifically, the same population of experimental units (somewhat ill-defined, as noted above) and the same treatments—Bayesian probability problems formatted in different ways. However, Gigerenzer and Hoffrage’s experiment is itself a conceptual replication of former experiments dealing with question of whether human inference naturally follows Bayesian principles. In this regard, Gigerenzer and Hoffrage (684) explicitly cite earlier experimental work by Rouanet (1961), Phillips and Edwards (1966) and Edwards (1968) that responded affirmatively to this question, as well as work by Kahneman and Tversky (1972, 1973) that responded negatively. Gigerenzer and Hoffrage’s idea is to propose a theoretical framework that can illuminate and resolve these conflicting pronouncements. The framework they utilize is evolutionary theory: they reason that if “humans have evolved cognitive algorithms that can perform statistical inferences”, then “as humans evolved, the ‘natural’ format was frequencies as actually experienced in a series of events, rather than probabilities or percentages” (686). As such their approach is to alter the treatments themselves, to modify the independent variables, by formatting the treatments as either frequencies or as percentages, as opposed to just operationalizing the original treatments in different ways. And, indeed, they discover that the participants are much more successful in solving these problems “in frequency formats” (684). It follows that Gigerenzer and Hoffrage, in subsequently showing that research participants are better at solving Bayesian problems expressed as natural frequencies, are able to generate an understanding of what was occurring in the former experiments in terms of providing a diagnosis for why in some of the earlier experiments human participants are successful, and in some of the other earlier experiments are unsuccessful, at effectively solving Bayesian probability problems. This is the sense in which a conceptual replication can have a value that goes beyond both exact and experimental replication.

However, it is Machery’s view that the notion of conceptual replication is ultimately confused. He maintains that “the usual typology of replications”, a typology that draws a contrast between direct and conceptual replications, is unprincipled since “a single type of replication corresponds to two distinct experimental components, while another type of replication corresponds to experimental units, [and] no replication corresponds to setting” (561–562).

In particular, the usual notion of a conceptual replication involves changes to either treatments or measurements, but neither changes to populations of experimental units nor to settings. This is what we saw with a number of examples of conceptual replication considered above. But that is not the case according to the definition of conceptual replication I have suggested here, since it is experimentally possible to replicate a concept or theoretical construct by changing any one of the four experimental components—one only needs a theoretical motivation to modify a component previously thought to be fixed. For example, in the experiments cited above concerning expectation effects experienced by humans, one might seek to determine whether rats, a different sort of experimental unit, experience expectation effects in their experiences with other rats, or whether humans experience expectation effects in different sorts of settings, such as when observing rats trained to press levers for food instead of running in mazes. This point is worth emphasizing since, according to Machery, the Resampling Account shows its superiority to other accounts by the fact that it treats experimental units, treatments, measurements and settings similarly in so far as “treatments or measurements can be sampled exactly as experimental units, and when one uses new stimuli or measurements (sampled from their respective populations) one does exactly the same thing as when examining new experimental units (e.g., new participants)” (559). By contrast, for him, conceptual replication fails in this regard and so is “confused” (560, 565). But this is not a flaw with conceptual replication, as I define it (nor indeed is it a flaw with how I define an experimental replication).

A further respect in which Machery believes his Resampling Account is superior to other accounts is that “on [his] account, not every experiment that is in some respect or other similar to an original experiment counts as a replication” (559). He does not provide much elaboration on this feature of his Resampling Account, but we can conjecture the following. He maintains that “the usual typology” of a conceptual replication leaves it unspecified “what a psychologist must do (resample, change the value of a fixed factor, etc.) to an experimental component for her experiment to count as a replication” (559). Now, with Machery’s Resampling Account, it is clear what counts as a replication: one replicates an experiment when one resamples the experimental components. On the other hand, it may be unclear whether we have a successful conceptual replication since the generated effect with a new experiment may be quite different from a previously generated effect—for example, with expectation effects, we are comparing behavioral responses to rats running mazes with similar responses concerning research subjects drinking coffee. Similarly, there may be a lack of clarity regarding whether a change in experimental components is suitable for a conceptual replication. So, for example, in Bargh’s ‘priming’ conceptual replication, being primed with pictures of Black males is compared to being primed with words indicative of elderly stereotypes, but it is not certain that these two kinds of treatments are sufficiently alike to constitute a form of replication. Accordingly, whether an experiment is a conceptual replication of another experiment is not a straightforward matter. It is a matter about which we need theoretical guidance in order for the notion of conceptual replication to be useful.

Still, despite this lack of clarity, it does not follow with the definition of a conceptual replication I am suggesting that every experiment similar “in some respect or other... to an original experiment counts as a replication”. There will be innumerable cases where experiments are similar to one another but do not constitute replications of one another. The same holds for what we have called an experimental replication. This is because, dependent on an experimenter’s theoretical understanding of the phenomenon under investigation, it may be a very specific matter what constitutes a conceptual or experimental replication, and there are changes to experimental components that will not lead to replications and in fact could amount to nonsense, even if the experiments are otherwise quite similar. For example, consider a purported replication of the original rats running-the-maze experiment where the only difference is that the replicated experiment is performed in a setting with an ambient temperature of − 25 °C. It's very similar to the original experiment, but certainly not a replication, given the confounding factor of an excessively low temperature.

One of Machery’s key reasons in support of his Resampling Account is that it grounds a principled distinction between replication and extension, in contrast with the usual notion of a conceptual replication which tends to conflate the two, such as with Schmidt’s (2009) account that “leads him to treat extensions as a distinct type of replication” (560). Machery’s definition of an ‘extension’ is as follows: it is either (1) “sampling from a different population (for the experimental components treated as random factors)” or (2) “changing the level of an experimental component treated as a fixed factor”. For Machery, “any acceptable account of replication must be able to draw this distinction” (560). And what is his definition of a replication? It is the process of resampling from a particular population, a population consisting of sets of experimental units, treatments, measurements and settings. Thus, we have a clear distinction between a replication which involves sampling from populations, as they are (either completely, with ‘fixed’ experimental components, or incompletely, with ‘random’ experimental components), and an ‘extension’ which involves changing populations and then sampling from them (again, either completely or incompletely). Now we might do the latter, that is, change the populations associated with experimental components, for a variety of reasons. For example, as we saw above, some methodologists are skeptical of the value of exact replication and argue that, to effectively test an experimental hypothesis, scientists need to operationalize in different ways certain variables or other experimental components. This process is often called conceptual replication, but I have alternatively described it as experimental replication as it does not involve a fundamental change to the variables. Alternatively, our motivation in changing experimental components might be to explore the promise of a theoretical perspective, such as evolutionary theory in Gigerenzer and Hoffrage’s experimental research, for the purposes of addressing an experimental problem. This is the strategy of what I am calling a conceptual replication, and it falls under the rubric of what Machery calls an extension. Indeed, an experimental replication as we have defined it is typically an extension too, since in re-operationalizing experimental components one modifies, as well, the population associated with these components.

There is, however, one kind of experimental replication that clearly isn't an extension, that is, an exact (or direct) replication. As Machery clarifies, “as a first approximation, a replication is direct if and only if it aims to be identical to an original experiment save for its sample of participants” (546). That is, on the usual view, an exact replication involves keeping everything about an experiment the same as far as possible, except that one resamples from a fixed population of experimental units. One way to understand Machery’s position is that, analogously to the usual notion of an exact replication, one can also have a form of ‘exact’ replication that samples not from a fixed population of experimental units but instead samples from fixed populations of treatments, measurements, or settings (these populations need to be fixed since if they change we have an extension, not a replication, on Machery’s view). Such an approach is not necessarily anathema to the traditional understanding of an exact replication. For example, Stroebe and Strack define exact replications as “replications of an experiment that operationalize both the independent and the dependent variable in exactly the same way as the original study” (2014, 60). Such a definition does not rule out the possibility that one exactly replicates an experiment by resampling from the populations associated with independent and dependent variables operationalized in a particular sort of way. In fact, such randomness is to be expected on the usual view since in the application of any treatment or measurement there is going to be some leeway in its deployment. For instance, in timing how fast rats run a maze, a method of operationalizing this treatment may involve a set of suitable measuring devices each of which can be used, but which are randomly and subtly different from one another. In general, the set of things associated with each experimental component, whether it be experimental units, variables, or settings, is going to be imprecise to some degree (just as we noted above concerning, specifically, experimental units). This imprecision is due in part to a certain degree of vagueness attending each of the components, so it can't be expected that experimenters will always associate with any experimental component the same population. But such flexibility points to a potential problem with Machery’s approach since, with a change in the population of experimental units, we no longer have a resampling, as Machery requires, but instead an extension. Thus, on the Resampling Account, it may be practically impossible to have true resamplings and any repeat of an experiment will inevitably be an extension. For this reason, Machery fails to provide a sustainable, principled distinction between a replication and an extension since surely, despite this variability, experimenters are able to perform replications. To be sure, Machery suggests that experimenters need to be expressly clear about the populations they are sampling from as regards experimental components to address the problem of imprecision (563–565). My point, however, is that, absent this further precision regarding populations from which experimenters sample, replications are nevertheless possible and have been performed for long time, Machery’s Resampling Account notwithstanding,

In arguing for the distinction between a replication and extension, Machery suggests that they have different functions: whereas “replications test the reliability of token experiments, extensions [test] their validity, as well as the invariance range of a phenomenon” (563). By the ‘reliability’ of a token experiment Machery means that, with replications of an experiment, the same result is generated “with high frequency” (555); by contrast, an experiment is ‘valid’ “just in case it actually supports the conclusion it claims to establish” (555). It's worthwhile pointing out here that Wells and Windschitl (1999), whom Machery cites as endorsing the view that experimental components other than experimental units need to be resampled, assert that stimulus sampling (that is, the sampling of treatments) is needed to avoid threats to ‘construct validity’. As they comment,

the use of only one stimulus to represent a category can confound the unique characteristics of the selected stimulus with the category. What might be portrayed as a category effect could in fact be due to the unique characteristics of the stimulus selected to represent that category (1116).

So, for these reasons, we might resist the assertion that replications (resamplings) test only the reliability of experiments and not their validity. Nevertheless, it is possible for one and the same experiment to both be reliable and valid. This could happen with exact replications insofar as they can be used for determining effect sizes and for ruling out the possibility of type one error—as indicated above, it has been suggested that hypotheses about effect sizes or about the absence of false positives can be confirmed by successful, exact replications. The same benefit accrues to experimental replications insofar as alternate operationalizations can reveal that an effect is robust, thus indicating that the effect is reliable in Machery’s sense, but where if the operationalizations used in testing an hypothesis’ robustness are suitably designed, such as to diagnose the presence of systematic error, a successful replication can also confirm the validity of an effect. Finally, a conceptual replication can be said to be reliable if, as guided by one’s theoretical understanding of a phenomenon, a previously found effect can be replicated. For instance, in showing that the research participants successfully solved Bayesian probability problems under a frequency formatting, Gigerenzer and Hoffrage reproduced the results of Rouanet (1961), Phillips and Edwards (1966) and Edwards (1968), where “inferences, although ‘conservative’, were usually proportional to those calculated from Bayes' theorem” (1995, 684). Similar conceptual replications, discussed above, reproduced the phenomena of ‘priming’ and ‘expectation effects’, thus confirming that these phenomena are real, as theoretically understood. Here, the benefit of conceptual replication is that these revealed phenomena are grounded in a theoretical perspective, one that is subject to independent verification. This is certainly the case with Gigerenzer and Hoffrage’s work where the motivating theoretical perspective is evolutionary theory, itself subject to substantial independent support. Hence, the reliability of experiments revealing a conceptually replicated effect speaks on behalf of the validity of this effect, as grounded in this theoretical support. Accordingly, Machery again fails to provide a principled distinction between a replication and an extension since experiments can be reliable and valid at the same time and in the same respect, in that their reliability—the fact that the same effect, as theoretically understood, occurs across different experiments—is the basis to the assertion of their validity.

I have argued, contra Machery, that the distinction between conceptual and direct application is (1) principled in that it adopts an even-handed approach to the four distinct kinds of experimental components, (2) is not confused so long as we distinguish between direct replications, experimental replications (sometimes called conceptual replications), and conceptual replications, as I define them, and moreover (3) illuminates the relationship between replication and extension—that is, sometimes they amount to the same thing.^2^ For my approach to work, however, it is important that the notion of a conceptual replication is clear. For example, one might suggest that the notion is too broad. There are many cases in science where convergent evidence is available in support of a theoretical hypothesis, but where we would not call such convergent evidence a form of replication. Here one might consider the various forms of convergent evidence that support the general theory of relativity, such as observations of gravitational waves, Eddington's eclipse experiments that demonstrated the bending of light in a gravitational field, and the successful prediction of the precession of Mercury's perihelion. One would not say that these forms of evidence constitute replications of each other. Does my approach suggest that they are?

When one is replicating an experiment, it is assumed that one is inventing a new experiment that is similar to the former experiment in many ways. The divergences could be quite small, such as with direct replication, where one is simply resampling a population of experimental units, treatments, measurements, or settings. Alternatively, as with experimental replications, the divergences might involve re-operationalizations of experimental components, without the components themselves being changed. Finally, with conceptual replications, one is modifying the experimental components themselves, but still exploring the same empirical phenomenon. In other words, the movement from exact to experimental to conceptual replications is gradational, with increasing divergences from the original experiment. Ideally, when one is performing an experiment, one first attempts an exact replication, likely for the reasons mentioned above, such as in establishing the existence of an effect or accounting for type 1 error. With an experimental replication, one strives to determine whether an exact replication is the result of a particular kind of operationalization of the experimental components. Finally, with a conceptual replication, one seeks to determine whether a similar phenomenon occurs in a related setting involving changes to the (fixed) experimental components themselves. In this regard, consider again the phenomenon of an expectation effect. We cited two experiments investigating this effect, the first studying expectation effects involving rats running in mazes, and the second exploring expectation effects with reference to people ingesting caffeine. In this case, psychologists will have in mind a theoretical perspective in which expectation effects can be found to occur in two distinct, but related settings, where the experimental components themselves are modified. The question, then, is whether an experiment involving research subjects exhibiting expectation effects when observing humans drinking coffee replicates a similar experiment in which research subjects exhibit expectation effects when observing rats running mazes. Again, on Machery’s view, the two experiments are not replications of one another since they involve alterations of fixed factors—they are, instead, extensions. Let’s suppose then that the rat/maze experiment works and that to all who appreciate the experiment it is clear that people have expectation effects about rats running mazes just as they have expectation effects about research subjects drinking coffee. It would follow that we have confirmed a particular theoretical understanding of an expectation effect, that it is an aspect of human nature that applies to both our experiences of rats and humans. In other words, it is confirmed that an expectation effect is one and the same phenomenon that can occur whether we study rats running mazes or study human research subjects drinking coffee. But it is hard to see how we could arrive at this conclusion if the experiments can't be said to be replications of one another.

By comparison, in a situation where we have convergent tests concerning the general theory of relativity, the different phenomena being examined are substantially different from one another and are not related by means of any progression of increasing generality, such as we find in the progression from exact to experimental to conceptual replication. The only tie that these tests of general relativity have to one another is that they are testing the same theoretical hypothesis. In this regard, recall once more Gigerenzer and Hoffrage’s experimental research. Here, the experimenters are not testing evolutionary theory but are attempting to resolve an experimental debate in the psychology of human reasoning. Since earlier experimental work had recorded conflicting results, Gigerenzer and Hoffrage utilize evolutionary theory to suggest (what I am calling) a conceptual replication of this work. In other words, they are not deploying a convergence argument in which conceptually diverse experimental results lead to the same conclusion, but instead are recommending a conceptually novel experimental strategy that replicates, but goes beyond earlier experimental approaches. The new strategy is a replication since it examines the same phenomenon as earlier work. By comparison, the diverse empirical tests cited for general relativity are not replications of each other as they involve different kinds of empirical phenomena unconnected by a progression from earlier exact and experimental replications. One could arbitrarily look at these tests as replications of one another, but that would be a stretch in terms of what experimenters typically take to be cases of replication.

## Conclusion

Replicating experimental work is of fundamental importance to science since scientific progress requires a stable empirical subject matter. This is why the reproducibility crisis has become so important to those who believe in the value of science.

Feest is correct that the problem of systematic error has the potential to undermine the value of replicability. It is of no benefit to have stable empirical results that are misleading. Yet, contra Feest, both experimental and conceptual replication have the resources is to address the problem of a systematic error. For example, by altering the operationalizations of experimental components one can ensure that a successful replication is not simply due to the nature of the operationalizations. Typically, these alternative operationalizations are formulated in advance of a replication, which allows us to address Feest’s concern that, when reflecting on successful replications, we beg the question against the possibility of a systematic error by proceeding ‘post hoc’. Moreover, Feest’s suggestion that experimenters focus more intently on identifying the sources of systematic error rather than engaging in replications is a nonstarter in so far as such an investigation itself requires access to a resource of replicable, empirical results.

For his part, Machery is correct that the usual typology of conceptual replication is to certain extent confused. To resolve this confusion I have suggested a distinction between what I call ‘experimental’ replication, which involves simply re-operationalizing experimental components, and ‘conceptual’ replication, which changes the experimental components themselves. The key point to keep in mind is that replication can involve more than just resampling, as Machery would have it. One can modify the components of an experimental process in various ways, either by keeping the variables the same while altering their operationalizations or by changing the variables themselves in strategic ways, and still be performing an replication since one is investigating the reality of a particular empirical phenomenon, such as the ability of a human to solve a Bayesian probability problem, the existence of social priming, or the occurrence of expectation effects, as opposed to extending one’s research to different sorts of phenomena. Also, given the imprecision inherent in determining what constitutes a population associated with an empirical component, it may be that (Machery’s notion of) resampling is unachievable in real experimental situations. If resampling is unachievable, it follows on Machery’s Resampling account of replication that every repeated experiment is an extension, which means we would no longer have replicable empirical facts on which to ground scientific progress.

## Footnotes

1 The sort of quandary that Feest attaches to conceptual replication is akin to what is historically known as the paradox of analysis—I cannot analyze what I do not know how to identify, and so analyze, already—and more recently was dubbed by Harry Collins the ‘experimenters’ regress’—that is, determining whether an experiment is reliable requires that I already have reliable knowledge of what the experiment is about.

2 The only case where they are definitely not the same thing is with direct replication.
